# Evolutions in Cardiovascular Implants—A Review of Past, Present, and Future

**DOI:** 10.3390/mi17060703

**Published:** 2026-06-08

**Authors:** Callen Moon, Jay Ming Tong, Dake Hao

**Affiliations:** 1 Department of Surgery, University of California Davis, Sacramento, CA 95817, USA; cdmoon@health.ucdavis.edu (C.M.); jaytong@ucdavis.edu (J.M.T.); 2 Department of Biomedical Engineering, University of California Davis, Davis, CA 95616, USA; 3 Shriners Children’s, Northern California, Sacramento, CA 95817, USA

**Keywords:** cardiovascular diseases, biomaterials, medical implants, tissue engineering, clinical applications

## Abstract

Cardiovascular disease (CVD) remains the leading cause of mortality worldwide, driving the continuous evolution of implantable cardiovascular therapies. Although early cardiovascular implants revolutionized the treatment of CVD, they are limited by restenosis, mechanical failure, poor biocompatibility, and inadequate tissue integration. These clinical limitations have driven the development of next-generation implants, with improved hemodynamic performance, regenerative potential, and long-term functionality. Advances in biomaterial science, tissue engineering, biosensors, wireless telemetry, flexible bioelectronics, and translational cardiovascular medicine have transformed cardiovascular implants from passive structural devices into biologically integrated and increasingly intelligent systems capable of interacting dynamically with the host cardiovascular environment. In this review, we summarize the historical evolution, current clinical applications, and emerging technologies of major cardiovascular implants. We further discuss key biological and engineering challenges limiting long-term clinical success and highlight future directions in regenerative biomaterials, smart bioelectronics, and personalized cardiovascular implants for next-generation cardiovascular therapy.

## 1. Introduction

Cardiovascular diseases (CVDs) remain the leading cause of death worldwide despite remarkable advances in pharmacological treatment, surgical intervention, and interventional cardiology [1]. The growing burden of ischemic heart disease, valvular dysfunction, heart failure, and peripheral vascular disease has driven the development of cardiovascular implants designed to restore physiological function and improve long-term patient outcomes [2,3,4]. Over the past several decades, technologies such as coronary stents, prosthetic heart valves, vascular grafts, cardiac patches, and implantable bioelectronic devices have transformed the clinical management of cardiovascular disease, establishing cardiovascular implants as a cornerstone of modern cardiovascular medicine [5,6].

The evolution of cardiovascular implants has been fundamentally driven by the diverse pathophysiological challenges presented by cardiovascular disease. Ischemic heart disease and atherosclerotic vascular occlusion stimulated the development of coronary stents and endovascular devices designed to restore vessel patency while minimizing restenosis and thrombosis [5]. Valvular heart diseases, including aortic stenosis and mitral regurgitation, motivated innovations in mechanical, bioprosthetic, and transcatheter valve technologies aimed at restoring hemodynamic function while balancing durability, hemocompatibility, and long-term structural integrity [7]. Cardiac arrhythmias drove the development of implantable pacemakers, cardioverter-defibrillators, and electrophysiologic mapping technologies capable of monitoring and restoring normal electrical conduction [8]. Myocardial infarction and heart failure encouraged the development of cardiac patches, regenerative scaffolds, and tissue-engineered constructs designed to repair damaged myocardium and promote functional recovery [9]. Vascular defects, aneurysms, and peripheral arterial disease further stimulated advances in vascular grafts and tissue-engineered vascular substitutes capable of restoring structural integrity and physiological blood flow [10]. Consequently, the evolution of cardiovascular implants can be viewed not merely as the advancement of individual devices, but as a continuous effort to address diverse pathophysiological processes and disease-specific clinical needs through increasingly sophisticated biomaterials, fabrication technologies, and bioengineering approaches. The relationships between major cardiovascular disease categories, their associated clinical demands, and representative implant technologies are summarized in Table 1.

**Figure 1:**
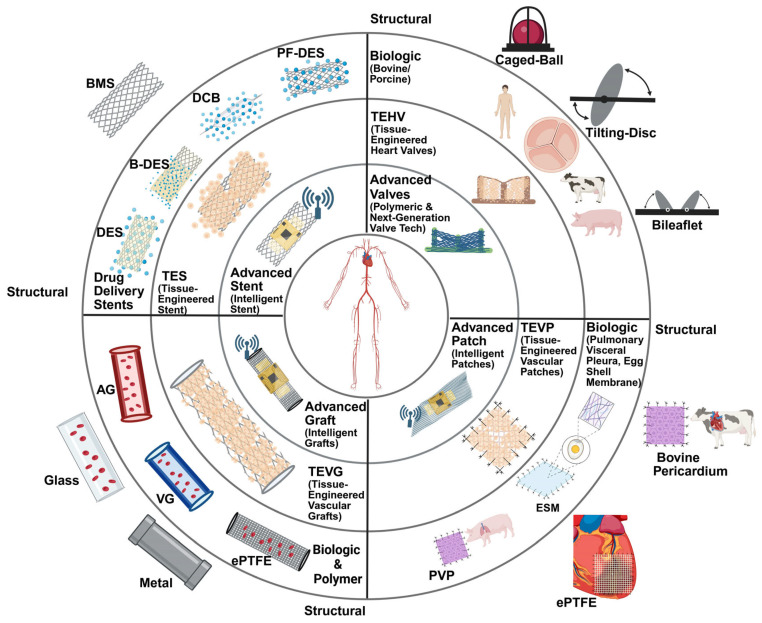
Evolution of cardiovascular implants. This diagram illustrates the generational advancements of cardiovascular implants, presented concentrically, progressing from early designs in the outer ring toward increasingly advanced implant technologies in the inner rings. Created in BioRender. Moon, C. (2026) https://BioRendercom/154nlp8.

The evolution of cardiovascular implants reflects the progressive advancement of biomedical engineering and translational cardiovascular medicine. Early-generation devices primarily focused on restoring anatomical and mechanical function, with limited understanding of thrombogenicity, inflammatory responses, immune-mediated rejection, biomaterial/host interactions, or long-term hemodynamic integration [11,12]. Although pioneering technologies such as bare-metal stents, caged-ball mechanical valves, and synthetic vascular grafts represented major breakthroughs in cardiovascular intervention, these devices were frequently associated with complications including thrombosis, restenosis, infection, calcification, structural degeneration, and compliance mismatch [13,14]. These clinical limitations highlighted the highly dynamic and biologically reactive nature of the cardiovascular microenvironment and ultimately drove the development of more biologically integrated implant systems. Advances in biomaterials science, tissue engineering, pharmacologic modulation, nanotechnology, and biofabrication have since redefined the design principles of cardiovascular implants. Drug-eluting stents (DES), bioresorbable scaffolds, polymeric and tissue-engineered valves, decellularized vascular matrices, and bioactive cardiac patches demonstrate the transition from passive structural devices toward regenerative and adaptive biomaterials capable of modulating inflammation, promoting endothelialization, and integrating with host tissue [2,15]. Simultaneously, emerging technologies in flexible bioelectronics, biosensors, wireless telemetry, and artificial intelligence have accelerated the development of “smart” cardiovascular implants capable of real-time physiologic monitoring, diagnostic feedback, and responsive therapeutic intervention [16]. Importantly, the evolution of cardiovascular implants has not occurred through isolated technological advances, but rather through iterative translational feedback between engineering innovation, mechanistic biological understanding, and clinical outcomes [17,18]. Failures and limitations of earlier-generation devices have continuously informed the development of next-generation implant architectures with improved biocompatibility, mechanical performance, and long-term functionality. As cardiovascular medicine increasingly moves toward personalized and precision-based therapeutics, the convergence of regenerative medicine, intelligent biomaterials, tissue engineering, and bioelectronics is expected to fundamentally reshape the future of cardiovascular intervention [19,20]. This progressive evolution from inert structural implants toward biologically integrated, regenerative, and intelligent cardiovascular systems is illustrated in Figure 1. In this review, we summarize the historical evolution, current clinical landscape, and emerging technologies of cardiovascular implants. We further discuss the major biological and engineering challenges that continue to limit long-term implant success. Finally, we highlight future directions in smart biomaterials, tissue-engineered systems, and biointegrated electronics that may enable the next generation of adaptive, regenerative, and patient-specific cardiovascular implants.

## 2. Pre-Implantation Diagnostics and Procedural Planning

The success of cardiovascular implants not only depends on device design but also on accurate diagnosis, anatomical characterization, and procedural planning prior to implantation [21]. Advances in cardiovascular imaging and catheter-based diagnostic technologies have altered patient selection, device sizing, and procedural techniques, which have substantially contributed to the clinical success of modern cardiovascular interventions [22]. Historically, cardiovascular diagnosis relied primarily on angiography and echocardiography for visualization of vascular and cardiac anatomy. The introduction of computed tomography (CT), magnetic resonance imaging (MRI), and three-dimensional image reconstruction substantially improved pre-procedural planning by providing detailed assessment of vessel geometry, plaque burden, valvular anatomy, and surrounding structures [23]. In coronary interventions, intravascular ultrasound (IVUS) and optical coherence tomography (OCT) enabled high-resolution visualization of plaque morphology, vessel dimensions, and stent expansion characteristics, improving lesion characterization and procedural precision [24]. Similarly, advanced CT imaging has become integral to transcatheter valve procedures through accurate annular sizing, coronary ostial assessment, and vascular access planning [25]. Although these technologies significantly improved anatomical assessment, structural imaging alone may fail to capture important physiological characteristics of diseased cardiovascular tissues. As cardiovascular therapies become increasingly personalized, there is growing interest in diagnostics systems capable of providing real-time electrophysiological, hemodynamic, and biochemical information before and during intervention.

Recent advances in flexible bioelectronics have expanded the role of catheter-based diagnostics beyond conventional imaging. Li et al. developed stretchable liquid-metal microelectrode arrays integrated onto balloon catheters capable of conformal contact with cardiac tissues during deployment [26]. These devices successfully performed both epicardial and endocardial electrophysiological mapping in canine and human heart models, generating high-resolution activation maps that enabled localization of pathological tissue regions through analysis of electrical propagation pathways [26]. The ability to obtain real-time electrophysiological information may improve procedural planning for cardiac patch implantation, arrhythmia mapping, electrophysiologic interventions, and future bioelectronic cardiac therapies by providing functional information not obtainable through anatomical imaging alone [26]. Beyond electrophysiological assessment, emerging catheter-based sensing platforms enable biochemical characterization of cardiovascular disease. Abiri et al. developed a balloon catheter-integrated electrical impedance spectroscopy (EIS) and electrical impedance tomography (EIT) system capable of identifying metabolically active atherosclerotic plaques through characterization of tissue electrical properties associated with oxidized low-density lipoprotein accumulation [27]. Using flexible microelectrode arrays integrated circumferentially around a balloon catheter, the investigators generated three-dimensional maps of oxLDL-rich plaques and demonstrated the ability to identify biologically active lesions that may be prone to instability and rupture [27]. Such capabilities may facilitate improved lesion selection, procedural planning, and risk stratification for coronary interventions by identifying vulnerable plaques before clinical manifestation [27]. The convergence of advanced imaging, physiological assessment, and flexible bioelectronic sensing reflects a broader transition from anatomy-guided intervention toward precision-guided cardiovascular therapy. Future diagnostic systems will likely integrate structural, electrophysiological, biochemical, and computational information into unified procedural planning platforms capable of supporting patient-specific decision making. As cardiovascular implants continue to evolve toward tissue-engineered, bioactive, and intelligent systems, corresponding advances in diagnostic and localization technologies will play an increasingly important role in optimizing device selection, implantation accuracy, and long-term clinical outcomes.

## 3. Stents

Coronary stents are expandable meshes used during angioplasty to prevent restenosis after restoring blood flow in the treatment of coronary artery disease. Evolution of coronary stents reflects years of progressive refinement of materials, methods, structures, and pharmacology. Each generational advancement sought to address the shortcomings of its predecessors alongside evidence-based guidelines to better serve patients with CVD. Evolving improvements in efficacy, restenosis, immunogenicity, and both short- and long-term complications acted as driving forces for the development of subsequent generations and continued to drive future research into the next generation of advanced smart stents (summarized in Table 2). Building from decades of refinement, the end goal is to achieve near-zero stent complications and incorporate modern biosensor technology to aid in the monitoring, prevention, and preemptive therapy of patients with CVD.

### 3.1. Structural Stents

At the basic level of stent structure there are two designs: self-expanding and balloon expandable. Stents were largely developed to overcome the shortfall of balloon angioplasty (BA), which included vessel dissection, vessel recoil, and high rates of restenosis [3]. The first bare-metal stents (BMS) were implanted in humans in 1986 (self-expanding) and 1987 (balloon expanding) and demonstrated greater therapeutic efficacy than BA by reducing restenosis and dissection [3,12]. BMS stabilize the vessel wall, and in doing so, make percutaneous intervention (PCI) more predictable and improve procedural success [3]. However, restenosis rates remained high due to extracellular matrix (ECM) deposition and smooth muscle cell proliferation, termed neointimal hyperplasia, as part of the inflammatory and proliferative response to endothelium damage [3,28,29]. This failure demonstrated the limits of purely structural BMS in supporting long-term vascular function. Despite their initial success, BMS have largely been replaced by DES except in specific scenarios and patient risk factors, such as contraindications to dual antiplatelet therapy (DAPT), which consists of aspirin and a P2Y12 receptor blocker [2,30,31]. BMS may also be considered in very large-caliber vessels or saphenous vein graft lesions where restenosis risk is intrinsically lower [3,30].

### 3.2. Drug-Eluting Stents

The first drug-eluting stents (DES) aimed at decreasing the rate of re-intervention after PCI due to relatively high rates of stent thrombosis seen in BMS via controlled release of immunosuppressant or antiproliferative drugs [12]. This pharmacologic function addressed the biological problem of restenosis. Despite their initial success in reducing neointimal hyperplasia, long-term safety is of concern due to late stent thrombosis thought to be due to the eventual loss of drug elution and remnant durable polymer coating and stent frame that acts as a nidus for thrombosis [12,15]. Improvements in polymer technology, pharmacologic dispersion kinetics, and drug delivery systems collectively establish the standard of care endorsed by the 2021 ACC/AHA/SCAI guidelines for nearly all PCI indications [2,15].

#### 3.2.1. Paclitaxel-Eluting Stents

Paclitaxel-eluting stents (PES) represent a milestone in the transition from purely structural stent scaffolds to pharmacologically active stents. Paclitaxel acts by binding microtubules and preventing their disassembly, thereby halting the cell cycle at the G0/G1 and G2/M transitions, and at higher doses promotes apoptosis [12,30]. This mechanism results in reduced restenosis rates by downregulating neointimal hyperplasia, reducing restenosis rates to under 10%, compared to the 20–30% restenosis rate of BMS [32]. Paclitaxel’s narrow therapeutic window and variability in tissue penetration raise concerns for toxic effects and variability in tissue healing, which can lead to long-term vascular complications [15]. Clinical studies demonstrated substantial reductions in restenosis compared with bare-metal stents, validating the concept of local antiproliferative drug delivery. However, concerns regarding delayed healing, late thrombosis, and the narrow therapeutic window of paclitaxel ultimately drove the transition toward limus family agents that offered improved safety profiles [15,31,33,34]. As a result, paclitaxel stents have largely been abandoned in favor of safer alternatives but represent a generational improvement that transformed stents from passive structural scaffolds into localized drug delivery platforms [31,32].

#### 3.2.2. Limus Family Drug-Eluting Stents

Sirolimus-eluting stents (SES) were the first of the limus family pharmacologic agents to receive regulatory approval, and early studies demonstrated significant reductions in restenosis [32]. Sirolimus and other limus family drugs (everolimus and zotarolimus) bind to the FKBP-12 intracellular protein and inhibit mTOR, a protein kinase [30]. This results in cell cycle arrest between the S/G1 phase and inhibits cellular proliferation without cytotoxicity [30]. Everolimus-eluting stents (EES) and zotarolimus-eluting stents (ZES) use derivatives of sirolimus with similar antiproliferative properties but with enhanced lipophilicity, allowing for more homogenous distribution throughout neointimal tissue, which reduces the risk of arteriosclerosis and restenosis [3,30]. EES show similar efficacy to SES with the additional benefit of reduced stent thrombosis [15]. These stents consist of a thin cobalt/chromium or platinum/chromium frame and everolimus-eluting fluorinated durable polymer [15]. These stents quickly became the benchmark for DES designs because they demonstrated improved flexibility and endothelial healing, which resulted in a 20% reduction in 5-year major cardiac adverse events, including stent thrombosis and target lesion revascularization compared to SES [35]. ZES, developed alongside EES, initially used a phosphorylcholine-based polymer with rapid elution of zotarolimus but saw unexpectedly high rates of stent thrombosis [15]. In response to these deficits, a hydrophilic polymer known as BioLinxTM replaced the phosphorylcholine-based polymer with the main goal of repulsion of thrombotic mediators due to hydrophobicity [15]. Comparative trials demonstrated that second-generation limus-eluting stents achieved similar efficacy in suppressing neointimal hyperplasia while improving safety compared with earlier DES platforms. These improvements were largely attributed to thinner struts, more biocompatible polymers, and optimized drug release kinetics that enhanced endothelial healing while maintaining antiproliferative efficacy [35,36]. The improved outcomes for EES and ZES over SES and PES demonstrate the evolution in stent design and polymer biocompatibility but are not without limitations. Though later revisions made the durable polymer more biocompatible, the residual polymer that remained on the stent struts after drug elution posed as a nidus for chronic inflammatory complications and serious cases of very-late stent thrombosis due to impairment of endothelial healing [15,32]. These challenges provided the rationale for further generations of DES, including biodegradable-polymer and polymer-free designs, which aimed to eliminate the permanent polymer exposure to the vessel wall.

#### 3.2.3. Biodegradable-Polymer Drug-Eluting Stents

Biodegradable polymers were engineered to address the chronic inflammation induced by residual polymer exposure. The polymer in these stents completely resorbs within months after implantation, leaving behind the bare metal scaffolding. This design preserves the beneficial antiproliferative effects of limus family drugs, while minimizing chronic vessel wall exposure to foreign material that can pose as a nidus for long-term stent thrombosis [15,32]. In addition, the scaffolding of biodegradable-polymer stents was made thinner which enhanced deliverability and flexibility [3,12,31]. This represents a shift toward materials that improve long-term biointegration. Valgimigli et al. [37] compared biodegradable-polymer sirolimus DES with durable-polymer zotarolimus DES in 1948 patients with acute coronary syndrome (ACS). Rates of major cardiac adverse events at one year were similar, demonstrating biodegradable-polymer DES was non-inferior but did not significantly reduce adverse outcomes. Though no significance was found, there was a numerical trend toward fewer late adverse events in the biodegradable-polymer DES, supporting the notion that polymer resorption may yield long-term safety benefits. Nicolas et al. [15] and Sabatine et al. [38] provide support for biodegradable-polymer DES and their ability to reduce long-term thrombosis, particularly in patients with high bleeding risk or small vessels. However, the latest durable-polymer DES already demonstrate exceptionally low complication rates, which makes it challenging to show absolute benefits necessitating usage of biodegradable-polymer DES over alternatives like EES and ZES [30,31]. This is echoed by current guidelines, which place biodegradable-polymer DES as an alternative to durable polymer DES, rather than as a replacement [37]. These findings align with the 2021 ACC/AHA/SCAI guidelines, which recommend individualized DAPT durations depending on bleeding risk [2]. EES and ZES are the preferred stents for most PCI procedures, demonstrating lower rates of thrombosis and restenosis than BMS and PES [35,36]. Guidelines recommend their use in stable coronary artery disease (CAD), ACS, diabetes, and complex lesions, including bifurcations and long lesions [2]. Their safety profile allows for wider application in patients at moderate bleeding risk, with flexibility in DAPT duration [15].

#### 3.2.4. Polymer Free Drug-Eluting Implants

Polymer-free DES were developed in response to concerns that even biodegradable-polymer might contribute to chronic inflammation and impaired vessel wall healing, especially in high-risk patients [39,40]. With polymer-free DES, the drug is applied directly to the BMS surface and is often contained within microporous reservoirs, which allows for continuous release without the use of polymers [41]. Early designs struggled with drug retention and homogenous drug penetration, and initial clinical outcomes fared worse than EES and ZES [32]. Newer platforms have refined drug-binding techniques to improve release kinetics, with strongest benefits postulated to be with high-risk bleed patients due to polymer-free DES requiring a shorter span of DAPT, usually consisting of 1–3 months post PCI, whereas traditional PCI requires 6–12 months [15]. This benefit is largely due to improved endothelialization of the stent, thereby reducing thrombotic risk [15]. Drug-coated balloons (DCBs) represent another polymer-free strategy where paclitaxel or sirolimus is delivered directly to the vessel wall during balloon inflation [15,42]. Following inflation, the DCB is removed and no permanent implant is left behind, which eliminates the risk of late-stent thrombosis and complications due to permanent scaffolding [42]. DCBs also confer the benefit of shortened DAPT, typically to 1 month or less following PCI, making them an additional therapy for high bleeding risk patients [42]. The BASKET-SMALL 2 trial demonstrated that paclitaxel-eluting DCBs were non-inferior to EES and PES in small-vessel CAD [42]. Further, these results showed similar rates of all-cause cardiac death and vessel or stent thrombosis within a 3-year follow-up period. Thus, there is difficulty in determining absolute benefits of DCBs over durable polymer DES due to the already exceedingly low complication rates. The latest advancements of drug-eluting implants include biodegradable-polymer and polymer-free designs that offer more personalized and safe solutions to coronary intervention. These stents are beneficial for high bleeding risk patients and where shortened DAPT (1–3 months) provides a patient safety benefit [2,37]. Drug-coated balloons (DCBs) have also emerged as a valuable option for in-stent restenosis and for small vessels where stenting may not be ideal due to higher restenosis rates [42]. These newer devices reflect a paradigm shift toward balancing efficacy, safety, and personalized therapy that best fits each patient and their comorbidity profile [3].

### 3.3. Tissue-Engineered Stents

Tissue-engineered stents (TES) are designed with the intention of providing a solution to late-stent thrombosis seen with DES by promoting endothelialization and improving tissue integration. Two methods exist: in vitro and in vivo. In vitro TES involve seeding autologous cells, such as human umbilical vein endothelial cells (HUVECs), endothelial colony-forming cells (ECFCs), human trophoblastic endovascular progenitor cells, and mesenchymal stem cells (MSC) onto the stent surface prior to implantation [43,44]. In vitro culturing complications included sloughing of the cultured cells upon implantation, spurring the development of modified stent coverings to promote cell adhesion [44]. However, depending on the method of attachment, blood supply to cells may be impeded such as in membrane-covered stents [44]. Methods such as grooved stent surfaces, nanotopographic metal surface modifications, and micropores have shown some benefit to endothelial cell adhesion to deter sloughing [44]. Similarly, ECM adaptations have been shown to improve cell adhesion through the use of matrix proteins such as collagen, fibronectin, and tropoelastin, with the additional risk of inducing host immune response due to activation against the matrix proteins [44,45,46,47]. Shi et al. [43] describe a preclinical animal trial of in vitro cell-seeded stents using endothelial progenitor cells (EPCs) bound to the stent scaffold with fibrin gels with some success, though their findings are limited by a lack of in vivo testing. In a similar manner, Wu et al. [41] demonstrated that MSC-seeded stents with gluten and polylysine ligands reduced in-stent restenosis in rabbits when compared to BMS. In vivo TES involve capturing circulating cells in the blood using linkers on a stent surface to promote endothelial regeneration [44,48]. Various antibodies such as anti-CD34, CD133, CD144, and VEGFR2 have been investigated for their potential to capture circulating ECFCs, though no cell-specific antibody has been found [44,48,49,50]. Additionally, the use of such antibodies in preclinical trials has shown poor support, often leading to no effect on neointimal thickness or endothelial regeneration attributed to cells other than circulating ECFCs [44,48,51]. Although largely still in the experimental phases, TES highlight the transition from static devices toward biologically active and interactive therapies that move PCI closer to true vascular restoration. If successful, TES could redefine stent therapy by reducing the chronic complications currently seen with DES and reduce the reliance on antiplatelet therapy, striving toward complete arterial healing.

### 3.4. Smart Stents

Seeking to expand the stent architecture beyond simply structural and pharmacological roles, smart stents seek to add diagnostic and therapeutic functionality for real-time monitoring of the vascular landscape. Engineering functionality into stents allows for measurement of pressure, flow, endothelialization, and restenosis, providing critical information for early intervention against complications [52,53]. Chen et al. [54] describe a balloon-expandable stent integrated with a micro-electromechanical systems (MEMS) sensor that measures pressure. This integration with a metal stent ingeniously uses the steel stent architecture as the RF antenna for the MEMS sensor, while upholding the structural function classically seen with steel stents. In porcine models this “smart stent” provided detection of restenosis and clot formation by measuring the pressure exerted against the stent frame, with a resolution of 12.4 mmHg [54]. This marks one of the early trials of smart stents and shows great promise for the future possibilities of biosensor technology integration with stent engineering. Musick et al. [55] expanded the role of smart stents by proposing the use of piezoelectric sensors for endothelialization monitoring via resonance frequency changes upon binding of endothelial cells to the stent surface. However, challenges with this proposition include the fact that normal healing may blur the sensitivity of the sensor to abnormal endothelialization, so future research should focus on sensor engineering to discern between normal endothelial healing and detection of abnormal endothelialization characteristic of restenosis. Monitoring blood flow and hemodynamics could also provide crucial information regarding post-PCI status. In addition, bioabsorbable platforms aim to combine temporary scaffolding with continuous biological monitoring to address both short-term and long-term complications traditionally experienced with PCI [53]. The promise of smart stents lies in transforming the stenting process from a stagnant implant to an active tool in cardiovascular care and monitoring. Future smart stents should emphasize bioresorption, hemocompatability, and devices capable of real-time wireless feedback. With this, there is also the potential for tailored drug delivery based on clinical parameters provided by real-time wireless monitoring. By embedding intelligence within stents, this generation aims to go beyond simply preventing restenosis by being able to predict and preemptively address adverse events before they occur. With this approach, cardiovascular care can be even further tailored to patient-specific cardiovascular care.

**Table 2:** Representative stent technologies, key innovations, clinical limitations, and translational progress.

Device/Material	Innovations	Clinical Limitations	Preclinical/Clinical Trials
Bare-Metal Stents	Stabilized the vessel wall and improved percutaneous intervention (PCI) success.	Neointimal hyperplasia led to high rates of restenosis.	BASKET-PROVE [31,33,34]
Durable Polymer Drug-Eluting Stents (DES) - Paclitaxel - Sirolimus - Everolimus - Zotarolimus	Reduced restenosis rates to less than 10% by releasing immunosuppressant or antiproliferative drugs.	Paclitaxel has a narrow therapeutic window and variable tissue penetration, leading to concerns about toxic effects and long-term complications. Limus family DES uncovered risk of late-stent thrombosis due to residual polymer causing chronic inflammation and impaired healing.	BASKET-PROVE [33] RESOLUTE ALL Comers [36] SORT OUT IV [35]
Biodegradable-Polymer DES Polymer-Free DES Drug Coated Balloons (DCB)	Addressed the issue of residual polymer induced thrombosis by using materials that fully resorb leaving a bare-metal scaffold. Polymer-free designs apply drug directly to stent surface. DCBs leave no implant behind.	Minimal clinical benefit compared to Durable Polymer DES.	Bioflow-DAPT [37] BASKET-SMALL 2 [42]
Tissue-Engineered Stents (TES) - In vitro - In vivo	Designed to address late-stent thrombosis by promoting endothelial regeneration. In vitro involves seeding autologous cells onto stent. In vivo involves stent modifications to capture circulating cells.	Challenges in experimental phases include sufficient cell adhesion and immunogenicity.	[41,43]
Smart Stents	Integrates diagnostic and therapeutic functions providing real-time monitoring of the vascular environment. Can also be tailored for drug delivery.	Inability to distinguish between normal and abnormal neointima. Concerns for sensor deformation and malfunction.	[52,54,55]

## 4. Heart Valves

Prosthetic heart valves have been used for decades to replace dysfunctional native valves and have undergone continuous improvement toward the ideal durability, hemodynamic efficiency, biocompatibility, and antithrombogenicity. Several types of heart valve prostheses include mechanical, biological, tissue-engineered, and tissue-mimicking valves. Unfortunately, current prosthetics demonstrate a “give and take” paradigm where, for example, biocompatibility and hemodynamic efficiency are compromised at the expense of durability. Thus, with each generation of valves there are trade-offs between various factors with no current prosthesis capable of offering all optimal characteristics. Though there are improvements between generations of valvular technology there is no single prosthesis that is universally optimal, which drives ongoing research toward the ideal prosthesis. The evolution of prosthetic valves is detailed in Table 3.

### 4.1. Mechanical Valves

Dr. Charles Hufnagel performed the first mechanical heart valve implantation in the descending thoracic aorta in 1952, which helped lay the groundwork for Dr. Dwight Harken in 1960 to successfully implant a caged-ball valve into the subcoronary position [11,56,57]. Since then, mechanical prostheses have been the cornerstone of valve replacement. In the time since the first caged-ball valves, only 6 of the 21 different valve designs are still in use [11,57]. These valves include the Starr–Edwards ball valve, Omniscience tilting-disc valve, Omnicarbon tilting-disc valve, Medtronic-Hall tilting-disc valve, St. Jude bileaflet valve, and Carbomedic bileaflet valve [11].

#### 4.1.1. Caged-Ball Valves

The Starr–Edwards caged-ball valve was developed in 1960 and consisted of a silicone ball within a polymethyl methacrylate cage. After undergoing several iterations, the SE#6120 mitral valve and SE#1260 aortic valve consisted of an alloy metal cage and heat-cured silicone ball which improved the durability of the valve [11,56,57]. However, it was bulky, had high transvalvular gradients, and produced turbulent flow that predisposed patients to several complications such as thromboembolism, hemolysis, and endocarditis [11,56,57]. Even paired with anticoagulation, the complications ultimately led to these valves being phased out by the 1980s [11,58,59]. Recipients of these valves still require follow-up care focusing on pannus formation and anticoagulation due to the valves’ long-lasting durability [56,58,60].

#### 4.1.2. Tilting-Disc Valves

The limitations of caged-ball valves led to the introduction of tilting-disc designs in the late 1970s. By incorporating a single-hinged disc that pivoted open with anterograde flow and closed with retrograde flow, near-laminar flow was achieved, which reduced turbulence and improved hemodynamics [11,56]. The Medtronic-Hall valve introduced in 1977, and modifications in 1979, increased the minor orifice diameter and changes in the pivot mechanism that allowed the disc to lift out of the housing and rotate, reducing areas of hemostasis and improving hemodynamics [58,61]. Other designs, such as the Omniscience valve, employed an off-center pivot to create one large and one small orifice, thereby reducing gradients, but inadvertently created stagnant flow prone to pannus and thrombosis [62]. The Omnicarbon valve, released in 1984, further refined the basic design of the Omniscience valve by replacing titanium housing with pyrolytic carbon, which improved material durability while retaining the single disc concept [11].

#### 4.1.3. Bileaflet Mechanical Valves

The St. Jude bileaflet valve, designed in 1977, was constructed with two semicircular leaflets that pivot on hinges to allow near-laminar flow, thus offering superior hemodynamics and lower risk of thromboembolism than the tilting-disc valves [11,56,57]. The valve was constructed of pyrolytic carbon, providing mechanical strength, wear resistance, and thromboresistance [58,62]. The bileaflet design minimized stagnant flow and turbulence, which translated to clinically lower thromboembolic risk and improved long-term outcomes compared to the caged-ball and tilting-disc valves [11,56,57]. Despite these improvements, the lifelong requirement of anticoagulation remains an important limiting factor, highlighting a trade-off between material durability and biocompatibility. Building on the success of the St. Jude valve, the Carbomedic valve introduced in 1986 was constructed from pyrolytic carbon and, similar to other carbon valves, demonstrated excellent durability and low mechanical wear. The Carbomedic valve featured a rotatable housing, allowing the surgeon to implant the valve ring and then rotate the valve leaflets to the desired position [57]. This meant the surgeon could align the leaflets parallel to blood flow direction to reduce turbulence, optimize effective orifice area, and improve hemodynamics. Thus, the Carbomedic valve was a durable alternative to the St. Jude valve with great long-term survival, low rates of valve-related mortality, and adjustability for surgeons who valued the rotation feature [63]. The On-X valve, constructed of pyrolytic carbon, represents the newest generation of bileaflet mechanical valves and features a flared inlet, elongated orifice, and 90-degree leaflet opening which optimizes laminar flow [64]. Most importantly, the improved features allow for less anticoagulation therapy, which can be titrated within the 1.5–2.0 INR range, demonstrated through the PROACT trial, compared to the 2.0–3.0 range needed for prior mechanical valves [58,65]. This positions the On-X valve as the current standard for mechanical bileaflet prostheses in both North America and Europe, particularly for patients seeking durability, such as younger patients or those who wish to avoid repeat operations [66].

### 4.2. Biologic Valves

The concept of bioprosthetic valves arose due to demand for alternatives to mechanical valves, which despite excellent durability, imposed lifelong burden of anticoagulation and carried high risk of thrombosis and hemorrhage [67,68]. Early efforts focused on replacing diseased valves with human donor tissue (homografts/allografts), pioneered by Donald Ross in the United Kingdom and Brian Barratt-Boyes in New Zealand in the 1960s. These cadaveric valves, either fresh or cryopreserved, preserved native anatomy and offered excellent hemodynamics but their use was limited by donor scarcity and progressive degeneration [69,70]. Almost concurrently, xenograft valves were developed by Alain Carpentier using porcine aortic valves mounted on synthetic stents, marking the first commercially available bioprostheses (Carpentier–Edwards and Hancock models) [70]. These xenograft models closely mimicked native valve geometry and improved hemodynamics compared with the bulky mechanical valves of the era. Early xenograft models suffered due to immune-mediated calcification and structure deterioration, which led to high rates of reoperation in younger patients [67,69]. To address these deficits, glutaraldehyde fixation techniques were developed that cross-linked tissue proteins, thereby reducing early rejection and antigenicity, and stabilized the leaflet structure, which provided a functional life of 10–15 years [70]. Subsequent anti-mineralization processes further improved biomaterial durability, but structural valve degeneration (SVD) remains the core functional limitation.

#### 4.2.1. Early Homografts and Xenografts

Homografts, also known as allografts, were some of the earliest bioprosthetic valves implanted. Ross & Barratt-Boyes derived these valves from human cadaveric aortic and pulmonary valves that were either fresh or cryopreserved [68]. Allografts provided excellent hemodynamics by preserving the native valve architecture and were particularly resistant to infection, making them preferred for cases of infective endocarditis [67]. Allografts were notably limited by donor availability, complex surgical procedures, and progressive degeneration over time that severely limited the lifespan of the valve [70]. Today, clinical use is severely limited, such as in the Ross procedure, where the patient’s own pulmonary valve is used to replace the malfunctioning aortic valve (autograft) and an allograft valve replaces the pulmonary valve [69,71]. Xenograft valves, or valves from other species, were first successfully performed by Carpentier–Edwards and Hancock and were constructed from whole porcine aortic valves mounted on a metal stent or polyester fiber (Dacron^®^) stents [67,69]. These valves preserved natural valve geometry and demonstrated near-physiologic hemodynamic performance superior to the mechanical prostheses available at the time [67]. However, early porcine valves had a severe deficit; durability suffered due to immune-mediated calcification and structural deterioration, which led to graft rejection and/or failure necessitating reoperation [68,69]. It was soon discovered that glutaraldehyde fixation offered some improvement of valve duration by stabilizing the tissue and reducing early immune rejection, but even with these refinements valve degeneration limited the valve lifespan to a median of 10–15 years [70].

#### 4.2.2. Porcine and Bovine Valves

Porcine aortic valve refinements and the introduction of bovine pericardial valves were developed in parallel. The Carpentier–Edwards Supraannular and Hancock II porcine valves incorporated design changes to reduce tissue stress and calcification. The Hancock II used a low-pressure glutaraldehyde fixation and anticalcification technique, while the Supraannular model increased effective orifice area and lowered gradients [70]. Bovine pericardial valves were introduced, such as the lonescu–Shiley valve and Carpentier–Edwards Perimount [58]. Unlike the porcine valves, the bovine pericardial valve consisted of pericardial tissue sutured to a synthetic stent and sewing ring, which allowed for flexibility in leaflet geometry and improved durability compared to porcine valves [64,68]. However, the Lonescu–Shiley valve was withdrawn due to tissue abrasion and mineralization that led to early valve failure [70]. By contrast, the Carpentier–Edwards Perimount proved more durable and became the model of pericardial valve technology for future valve design [58]. Despite these improvements, structural valve degeneration remained inevitable, especially in younger patients with more robust immune systems, where up to 45% of patients under 40 years old required reoperation within 15 years [69]. Consequently, these valves were most suited to older patients with contraindications to anticoagulation [57]. Designs such as the Carpentier–Edwards Perimount Magna Ease, Trifecta, and Inspiris Resilia continued to expand upon prior technology and incorporated thin bovine pericardial sheets mounted on flexible stents, which optimized leaflet motion [58]. Thinner leaflets allowed for better hemodynamics, and new sewing ring designs reduced pannus formation [58,72]. In addition, proprietary anti-mineralization processes that targeted calcium-binding phospholipids and aldehyde residues improved valve durability but degeneration still persisted with an average valve lifespan of 10–15 years, especially in younger patients [58,70,72]. However, these bioprostheses became the standard for elderly patients and aided in the development of transcatheter aortic valve replacement (TAVR), which uses similar materials [69].

#### 4.2.3. Novel Xenografts and Bioprostheses

Recent designs integrate biologic and synthetic strategies to extend durability and reduce immune-targeted valve degeneration through stentless and sutureless valve designs, as well as advanced tissue engineering. Stentless valves such as the Toronto SPV, Medtronic Freestyle, and Sorin Freedom Solo eliminate the rigid stent, which improves hemodynamics at the cost of reducing durability and increasing surgical complexity, which may lead to higher rates of reoperation when compared to stented valves [57]. On the other end of the spectrum, sutureless valves such as the Perceval S and Intuity Elite allow for rapid deployment and reduced bypass and cross-clamp duration, which provides benefit for patients where minimally invasive procedures are preferred and in high-risk patients [57]. By understanding the fundamental limitations of bioprosthetic valves and the pathophysiology behind structural degeneration and calcification, the next generation of bioprosthesis can target the underlying limiting factors. One promising avenue is the use of genetically engineered xenografts which are derived from pigs lacking the key xenoantigens thought to cause immunologic response [69]. Genetically modified pigs with triple knockout genes making lack the xenoantigens α-galactosyl epitope (α-Gal), N-glycolylneuraminic acid (Neu5Gc), and Sda antigen, reducing immune recognition and subsequent calcification [69]. These valves provide hope that valve life could extend beyond the current 10–15-year lifespan and potentially make prosthetic valves more feasible for younger patients where current guidelines recommend mechanical prosthesis. Simultaneously, tissue-engineered valves combine decellularized xenograft scaffolds with bioactive strategies such as stem cell seeding, surface coating, or cross-linking chemistries with the aim of providing regenerative capacity, promoting host endothelialization, and reducing immunogenicity [68]. Although genetically modified bioprosthesis and tissue engineering valves are still in the experimental stages, they nonetheless represent a shift toward overcoming the limitations of prior valve generations with bioactive and patient-specific valve substitutes.

### 4.3. Polymeric Heart Valves

The primary goal driving polymeric valves lies in the desire to overcome limitations of mechanical and biologic valves. Mechanical valves, though durable, require lifelong anticoagulation. In contrast, biologic valves offer superior biocompatibility and less stringent anticoagulation requirements, but are limited by structural degeneration and calcification [64,68]. Advances in polymer research have opened the possibility of constructing synthetic valves and valve components that resist calcification, thrombosis, and degeneration while also offering favorable hemodynamics and, more recently, minimally invasive valve replacement methods.

#### 4.3.1. PTFE, Polysiloxanes, PU and PET

The first polymer-based valves date back to the 1950s, with the first animal studies beginning in 1960 using a polyurethane valve [4]. However, these valves had poor durability, leaflet tearing, and calcification when faced with physiologic stress, which made early polymer valves vastly inferior to the rapidly advancing technology of mechanical and bioprosthetic valves of the time [4]. Other materials such as polysiloxanes, polytetrafluoroethylene (PTFE), poly (L-lactide-co-ɛ-caprolactone (PLCL), poly (lactic-co-glycolic acid) (PLGA) and poly (L-lactic acid-hydroxyacetic acid) (PLLA) also had poor results and demonstrated poor biocompatibility and high calcification rates [4]. Early iterations of PTFE and ePTFE suffered due to hardening and calcification of leaflets and poor initial implantation outcomes [4]. Their reduced flexibility led to increased valve stress, resulting in higher rates of degradation [73]. However, substantial advancements have been made, such as increased flexibility and micropores, which allow ePTFE to be primarily used for congenital heart disease repair, including pulmonary valve and right ventricular outflow tract obstruction (RVOT). These valves provide excellent biocompatibility, low infection risk, calcification resistance, reliable durability, and readily available synthetic materials that are cost-effective and able to be tailored to patient-specific anatomy [74,75]. In comparison, homografts and bovine jugular vein conduits are also commonly used, though these valves suffer due to early degeneration, calcification, and increased infection rate (11.3% homograft vs. 0.3% ePTFE), making their long-term viability subpar [75]. Polysiloxane valves are silicone based and offer excellent biostability, biocompatibility, and wear resistance. However, in early non-human trials, long-term survival suffered and early human programs were discontinued due to high mortality rates [4,76]. Although subsequent modifications and animal trials showed some promise, their durability varied and silicone valves have largely been phased out without large-scale human trials [4]. Polyurethanes, like other valve polymers, suffered from insufficient durability in the early stages of experiments. PU compounds contain degradation-susceptible bonds via hydrolytic and oxidative degeneration, thus producing leaflet cracks and increasing fragility limiting valve life to 1–3 years [76]. Polyethylene Terephthalate (PET/Dacron^®^) has been widely used in cardiovascular prosthetics since 1957, specifically in vascular grafts, due to good mechanical properties such as strength and tensile stability [4]. However, these same strengths in vascular grafts were weaknesses when applied to valve construction because rigidity led to abnormal hemodynamics, accelerated leaflet degeneration, and cusp tearing [77]. Li et al. [77] explain the failures seen with polymer valves as a need to shift valve development toward matching the biologic and biomechanical environment of the heart. Much work has been conducted to improve biocompatibility, decrease immunogenicity, and improve hemodynamics, yet accelerated rates of valve degeneration and mechanical failure remain critical challenges. The first generation of polymer valves were developed as homogenous, isotropic, linear elastic materials, which are in stark contrast to the hierarchical, anisotropic architecture of collagen and elastin that make up native valves. This biomechanical mismatch means first generation valves could not replicate the native leaflet’s stress–strain behavior and explains the accelerated degeneration, cusp tears, delamination, and calcification.

#### 4.3.2. POSS-PCU, Silicone, SiPUU, PVA, SIBS and FGO-PCU

The limitations of the first-generation polymer valves prompted innovation in the 1990s and 2000s aimed at advancing polymer chemistry, nanotechnology, and composite materials that led to several promising materials. Promising materials include polyhedral oligomeric silsesquioxane polycarbonate-urethanes (POSS-PCU), polysiloxane-polyurethane ureas (SiPUU), polyvinyl alcohol (PVA), styrene-isobutylene-styrene (SIBS), and fluorinated graphene oxide-reinforced PCU (FGO-PCU) [4,76,77,78]. POSS-PCU is a nanocomposite material consisting of POSS nanoparticles incorporated into flexible PCU and covalently bonded to a hard aromatic compound segment [77]. This combination showed improved oxidative resistance, reduced calcification, enhanced tensile strength, and improved durability to a valve life of 15–25 years [76]. Small animal studies have demonstrated reduced thrombogenicity compared to first-generation polyurethanes, though large-scale human data remain limited [4]. SiPUU experimental valves also incorporate a soft segment for flexibility and a hard segment, which increases durability and oxidative stability by incorporating siloxane segments into the PU chain [76,79]. Advancements in SiPUU laid the groundwork for the LifePolymerTM material used in the Foldax^®^ TriaTM valve, which is the first polymeric valve to progress into human clinical trials [4,78]. Ongoing clinical trial results in low-risk TAVR patients are promising and demonstrate excellent deliverability, hemodynamics, antithrombogenicity, healing, and clean leaflets [78]. PVA hydrogels are hydrophilic polymers with great biocompatibility and low thrombogenicity, and also offer seemingly unlimited tunability [78]. PVA tensile properties can be adjusted by subjecting the polymer to freeze–thaw cycles while strength/rigidity can be adjusted via physical cross-linking [78]. However, there are currently no clinical trials or studies showing long-term in vitro or in vivo compatibility. SIBS, which has successfully been used in stents, offers excellent resistance to hydrolysis, oxidation, and other enzymatic cleavage reactions that other polymeric valves are susceptible to [78]. However, the hardness of the material makes it particularly susceptible to creeping, which is repetitive stress placed on the valve by the heart that causes incremental narrowing of the valvular lumen leading to stenosis or stress-cracking [78]. Fiber reinforcement experiments showed improved creep resistance but in vivo tests showed destruction of both the polymer and fiber [78]. xSIBS, developed by Innovia LLC, is a thermoset polymer that improves durability without fiber reinforcement while also offering flexibility and tear resistance; it later became the model polymer for the Polynova TAVR valve [78]. In the Polynova TAVR valve xSIBS demonstrated great antithrombogenicity but suffered in comparison to the Carpentier–Edwards PERIMOUNT Magna Ease due to high regurgitation (6.9% Polynova vs. 1.11% PERIMOUNT), likely due to creep and stress fractures [78,80,81]. Thus, additional research and testing are needed to improve the mechanical properties of SIBS and xSIBS to better match the mechanical stressors of the heart. FGO-PCU (Hastalex) is a nanocomposite that incorporates graphene oxide nanosheets that provide excellent tensile strength, fatigue resistance, hydrophobicity, and reduced thrombus formation. In addition, the strength of the polymer allows for production of thinner leaflets which can be used to improve flexibility [78]. Though there is promise that FGO-PCU is more resistant to calcification and has better biocompatibility when compared to ePTFE and bovine-pericardium, there are no data on in vivo testing and hemodynamic performance of FGO-CPU based valves [78]. Overall, second-generation polymeric valves mark a turning point in polymeric valve research. Though many of the materials discussed are still experimental, they represent the trend toward valves that are better matched for the mechanical and biological environment of the heart. Still, human outcome data and clinical validation are sparse, and no polymer valve has achieved regulatory approval.

### 4.4. Tissue-Engineered Valves

Unlike their predecessors, tissue-engineered heart valves (TEHVs) are designed for the regenerative function of long-term valves, which is critical for growing patients [13]. The advancements of TEHVs move us closer toward the “ideal” valve, with special consideration for the benefit of pediatric populations where repeated operations remain a large clinical burden because current valves are not capable of growing with the patient [13]. Several methods have been proposed and investigated, including in vitro engineering, in situ tissue engineering, and decellularized matrices. Early TEHVs consisted of a biodegradable scaffold seeded with autologous cells. Several limitations were associated with this approach, including time required for culture, scalability, and cost. In vitro culture can take weeks to months before implantation, which severely limits use in emergency applications [13]. Adding to complexity, the valves must be seeded with autologous cells from the patient to avoid immune rejection [13]. These obstacles are not only expensive but also limit the scope of patients that this solution may be viable for because of the time necessity. Regardless, in vitro valves from the perspective of functionality represent an advancement in antithrombogenicity and hemodynamic stability [13]. A more viable and applicable alternative is in situ tissue engineering, where rather than autologous seeding of replacement valves, the implant relies on host mediated regenerative potential. In situ valves are acellular and provide a bioresorbable scaffold off-the-shelf that relies on the host body to populate and remodel the valve. Though novel in theory, the lack of controllable neoproliferation and ECM deposition has been shown to cause valve failure and leaflet thickening within 12–24 weeks of implantation [13]. Decellularized matrices offer another alternative approach with promising results. Using this method, natural valve tissue from human donors or porcine/bovine sources is processed to remove all the donor cells [82]. This leaves behind a decellularized structure consisting of ECM that, when implanted, is infiltrated by the hosts cells to repopulate the scaffold [70,83]. This method removes immune rejection risk, provides “off the shelf” availability, and can be implanted via transcatheter [13]. The ARISE trial followed 144 patients (median age of 30.4) who received non-cryopreserved decellularized aortic homografts (DAH) for aortic valve replacement [84]. In this trial, they showed comparable results between DAH and Ross procedures, 95.7% 10-year survival, and excellent hemodynamic function [84]. However, 10-year functionality decreased, particularly in the pediatric subset of the trial (*n* = 40), which is attributed to increased immunogenicity to peptides found in the ECM as a result of the decellularization process [84]. The successes of this trial highlight how crucially important understanding of the immunogenic response is to furthering progress toward the ideal valve [69]. TEHVs center around active regeneration, scalability, and off-the-shelf simplicity. Despite continued challenges, decellularized valves demonstrate a hopeful trajectory toward the ideal valve, as shown through strong clinical trials and outcomes [82]. Though immunogenicity remains a barrier, current advancements highlight both the progress made and future obstacles that lie in the path toward the ideal valve [69]. In addition, post-implantation ischemia of valve tissue remains a key limitation due to the lack of microvascularization, leading to valve failure [82]. Thus, future work is necessary to improve the vascularization of TEHVs to support tissue growth.

**Table 3:** Representative prosthetic heart valve technologies, key innovations, clinical limitations, and translational progress.

Device/Material	Innovations	Clinical Limitations	Preclinical/Clinical Trials
Mechanical Valves - Caged Ball - Tilting-Disc - Bileaflet - On-X Valve	Were the first successful valve implants and have great durability.	All mechanical valves require lifelong anticoagulation therapy, which carries a high risk of thrombosis and hemorrhage.	PROACT [60,61,63,65,66,85]
Biologic Valves - Homografts - Xenografts - Stentless - Sutureless	Homografts preserve native valve architecture and thus have great hemodynamic performance. Xenografts mimicked native valve geometry and were less bulky than mechanical valves. Stentless valves eliminate the rigid stent to improve hemodynamics. Sutureless valves allow for rapid deployment in minimally invasive procedures.	All are limited by structural deterioration and calcification often within 10–15 years of implantation. Homografts are limited by donor availability and complex surgical procedures such as the Ross procedure.	[59,71,72,84,86,87,88,89,90,91,92,93,94,95,96,97]
Tissue-Engineered Valves (TEHVs) - In vitro - In vivo	TEHVs use decellularized matrices that can be repopulated by host cells, offering low immunogenicity and the potential for off-the-shelf options. May also be useful for pediatric patients where the valve can grow with the patient.	In vitro methods are time consuming and not suitable for emergencies. In vivo valves lead to valve failure due to uncontrolled neoproliferation. Decellularized valves still face challenges of longevity due to immunogenicity of extracellular matrix proteins, particularly in patients with strong immune systems.	ARISE [84]
Tissue-Mimicking/Polymer Valves - POSS-PCU - SiPUU - ePTFE - FGO-PCU - SIBS & xSIBS	New materials with improved oxidative resistance and durability in addition to innate biocompatibility of synthetic materials.	Early polymer valves tore leaflets, calcified, and degenerated faster than expected.	[4,73,74,75,79,81,98]

## 5. Vascular Grafts

Vascular grafts form the basis for several procedures in cardiovascular surgery, including coronary artery bypass graft (CABG) surgery, peripheral arterial revascularization, hemodialysis access, and repair of congenital malformations. Autologous grafts such as the saphenous vein and internal mammary artery (IMA) remain the gold standard due to natural biocompatibility and superior long-term patency, but their availability can be limited by prior harvest, comorbidities, or anatomical differences [99]. Thus, the need for alternatives drives the development of synthetic variants, including Dacron^®^ and expanded polytetrafluoroethylene (ePTFE, Gore-Tex^®^). The progression of these grafts is outlined in Table 4. These materials demonstrate high efficacy in large-diameter applications (>6 mm) with a 5-year patency rate around 90% [100,101]. However, they consistently fail in small-diameter applications (<6 mm) due to thrombosis, intimal hyperplasia, and compliance mismatch [100,101,102,103]. Recent advances in tissue engineering technology focus on developing small-diameter grafts using decellularized ECM, biohybrid scaffolds, and regenerative approaches. Despite promising results, no synthetic tissue or tissue-engineered small-diameter graft has yet matched the long-term patency and clinical reliability of autologous grafts. As such, this is the driving force for current research goals toward a clinically viable, off-the-shelf small-diameter graft that overcomes current limitations [14]. The following sections highlight the history and development of each generation of vascular grafts.

### 5.1. Structural Vascular Grafts

The earliest attempts at vascular replacement employed rigid, non-biological materials such as glass, ivory, and metal tubes which unsurprisingly failed due to thrombosis, turbulent flow, lack of endothelialization, poor hemocompatibility, and infection [104]. The next advancement came in 1952 where surgeons noticed a film of endothelial tissue covering woven sutures in a right ventricle where the fabric was consistently exposed to blood flow [104]. This observation sparked the idea for use of synthetic materials and polymers such as nylon, Orlon, polyurethane, and Dacron^®^ in vascular repair where the porous nature of the material allowed for the formation of stable adherent thrombus and fibrous neointima that mimicked the vessel’s native luminal surface [104]. This marked a major shift in prosthetic graft design toward porous materials that enabled tissue integration, driven by the observation of neointimal hyperplasia in earlier smooth, impermeable conduits [104]. Despite these innovations, clinical outcomes were largely dependent on usage. Grafts over 8 mm in diameter performed well, while grafts with diameters smaller than 6 mm failed due to thrombosis and intimal hyperplasia [100,101]. These failures emphasized the need for biocompatibility and showed that mechanical success was not sufficient in and of itself. The use of Dacron^®^ (PET) and ePTFE became the materials of choice with the highest efficacy. Dacron^®^ could be woven or knitted, and each variant had different clinical applications. Woven Dacron^®^ provided durability and impermeability, but was less flexible [105]. In comparison, knitted Dacron^®^ was flexible but less durable, and required preclotting to prevent plasma leakage due to higher permeability [105]. To overcome the disadvantages of knitted Dacron^®^, collagen, albumin, or gelatin coatings were added to reduce the permeability while retaining the flexibility [105]. Dacron^®^ became the material of choice for aortic and iliac grafts, largely due to the success in large diameter settings (>6 mm) where 5-year patency approached 90% [100]. However, outcomes remain poor in femoropopliteal and coronary bypasses, with an estimated 71–75% 5-year patency rate [100]. ePTFE (Gore-Tex^®^) also provided promise for medium-sized vessel grafts due to its microporous structure that permitted tissue ingrowth while maintaining flexibility, sterility, and low thrombogenicity [106].

### 5.2. Autologous Arterial and Novel Vein Grafts

Despite issues in anatomical availability, autologous grafts such as the IMA and radial artery (RA) remain the gold standard for vascular reconstruction due to their long-term patency rate exceeding 90% at 10 years [107]. Though autologous grafts have superior attributes, including native endothelial integrity, elasticity, and atherosclerosis resistance, some grafts are not feasible for all applications [107]. For example, the saphenous vein offers easy harvesting and is widely available, but the high compliance of the vein renders it unsuitable for arterial circulation due to higher systemic pressure and shear stress [108,109]. Thus, though venous autografts are more readily available, they often have lower 10-year patency rates (50–60%) in arterial applications than artery autografts due to endothelial injury, intimal hyperplasia, and ultimately graft failure [108,109]. To overcome the scarcity of autologous tissue options, Herrmann et al. [108] developed cryopreserved autologous vein grafts (CAVGs) for CABG in patients where native graft options are infeasible. In this study, saphenous veins were de-endothelialized and cryopreserved. This study, though limited to only 32-month patency, provided evidence of physiologic adaptation in autologous scaffolds. Histological findings suggest collagen remodeling and endothelial regeneration, demonstrating the ability of the graft to heal. Autologous arteries remain the benchmark, but with limitations in availability, CAVGs represent an important alternative for small-vessel grafts as an off-the-shelf format with reproduction of native vessel compliance, antithrombogenicity, and remodeling capacity.

### 5.3. Tissue-Engineered Vascular Grafts

The limitations of both synthetic and hybrid grafts in their inability to achieve long-term patency in small-diameter vessels provided the driving force for the development of tissue-engineered vascular grafts (TEVGs). These grafts were developed to be living and dynamic structures that adapt, repair, remodel, and grow similarly to native vessels. TEVG design includes three interdependent components: scaffolds, cells, and signals [103]. Scaffolding provides initial mechanical durability for the graft along with a template for neotissue to follow. Scaffold material varies and can be made from a range of synthetic, natural, and decellularized xenograft/allograft matrices [103,110]. The seeding of biodegradable polymer scaffolds with autologous bone marrow-derived mononuclear cells by Shinkoka et al. [111] marks a major breakthrough as these grafts demonstrated organized neointima, patency without rupture or aneurysm at 7 years, and adaptation to growth in children [101,103]. The study by Olausson et al. [112] describes the use of decellularized allogenic grafting of a human iliac vein seeded with stem cells to graft between the superior mesenteric vein and intrahepatic portal vein in a 10-year-old girl. Complete recellularization occurred 2 weeks after implantation and at the time of publication there was no evidence of anti-EC antibodies, and the patient did not receive immunotherapy. Though there were some complications due to mechanical obstruction, the graft remained patent after resection of mesocolon tissue causing the obstruction. Olausson et al.’s study marks one of the few examples of the use of TEVGs in humans and provides promise for clinical translation. Development of pulsatile-flow bioreactor systems improved tissue development and resulted in engineered vessels with compliance comparable to native vessels [101]. Furthermore, the development of decellularization provided human acellular vessels (HAVs) composed of remnant ECM structure and off-the-shelf viability. The study by Lawson et al. [113] describes a phase 2 clinical trial for dialysis access using HAVs and demonstrates high patency rates, infection resistance, and capacity for host recellularization. Primary patency, defined as vessels not requiring any intervention, was 63%, 38%, 18%, and 15% at 6, 12, 18, and 24 months, respectively. However, secondary patency, defined as functional access patency, showed patency of 97%, 89%, 81%, and 80% at 6, 12, 18, and 24 months, demonstrating a promising grafting method. Although HAVs appear safe and durable, long-term patency beyond 24 months, scalability, and standardization remain unproven. However, larger trials and additional testing are necessary to evaluate the efficacy of these grafts [14]. In contrast to in vitro pre-seeding, in situ endothelialization uses the human body’s own ability to repopulate the vascular scaffold [14,114,115,116]. This is usually accomplished by engineering ligands that bind to circulating endothelial progenitor cells, which enables off-the-shelf usage, as opposed to the complex and time-consuming logistics of pre-seeded scaffolds that require decellularization and stem cell culture [14,117]. Although currently only in animal trials, in situ endothelialization may enable higher clinical translation potential compared to in vitro TEVGs.

### 5.4. Smart Grafts

Beyond structural, anti-immunogenic, and hemodynamic advancements lies the opportunity for real-time diagnostics, remote monitoring of the vascular landscape, and therapy titration via smart grafts, facilitating timely intervention before critical graft failure. Recent prototypes integrate flow biosensors that provide continuous hemodynamic monitoring capable of distinguishing changes in pulse, pressure, and thrombi detection in vitro [16]. Additionally, biosensors have been shown to monitor clot dissolution during thrombolytic therapy [16]. Integration with wireless electronics also enables remote monitoring, providing a viable option for postoperative surveillance and telemedicine applications [16]. Although these technological advancements are still in preclinical development, in vivo implantation in rabbit carotid arteries has demonstrated robust dynamic stability under bending, compression, and twisting forces [16]. Thus, smart grafts represent the convergence of material science and digital health by integrating dynamic biointerfaces with structural grafts to address challenges such as late-stent thrombosis via continuous post-implant monitoring. Further innovation can seek to add drug reservoirs into vascular conduits, allowing for real-time drug administration in sync with biosensor monitoring.

**Table 4:** Representative vascular graft technologies, key innovations, clinical limitations, and translational progress.

Device/Material	Innovations	Clinical Limitations	Preclinical/Clinical Trials
Purely Structural - Glass & Metal - Dacron^®^ - ePTFE (Gore-Tex^®^)	Dacron^®^ and ePTFE were developed after the failure of glass and metal grafts. ePTFE and Dacron^®^ are porous and allow for neointimal proliferation that mimics the native vessel lumen.	Consistent thrombosis and intimal hyperplasia in small vessel applications.	[99,100,105,106,118]
Autologous - IMA - RA - CVG - CAVGs	Patient-derived conduits are benchmark (>90% 10-year patency). CAVGs extend feasibility for patients lacking autologous options.	Limited anatomical availability, SVGs prone to intimal hyperplasia and 50–60% 10-year patency; CAVGs show short-term patency (32 months) and require complex preparation.	[107,108,109]
Tissue-Engineered Vascular Grafts (TEVGs)	Designed to adapt, repair, and grow, TEVGs offer an off-the-shelf vascular graft using human acellular vessels (HAVs).	Few human implantations. Long-term patency and standardization of HAVs are unproven and remain experimental.	[110,111,112,113,119]
Smart Grafts	Integrates biosensors for real-time monitoring, such as pressure, pulse, and thrombogenesis. Holds promise for detection of complications through continuous post-implant surveillance.	Still in experimental stage and lacks evidence supporting viability.	[16]

## 6. Patches

Patches are sheets of biologic or synthetic material that may be used in procedures such as arteriotomy closure, endarterectomy, angioplasty, and congenital cardiac repairs. The development from structural to smart patches is presented in Table 5.

### 6.1. Structural and Clinical Benchmarks

Purely structural patches include bovine pericardium, ePTFE, and Dacron^®^ and provided mechanical reinforcement to the site of arteriotomy. Bovine pericardium became widely adopted due to availability but suffers from calcification and lack of remodeling, growth adaptation, or regeneration [120]. Other alternatives such as ePTFE and Dacron^®^ offered off-the-shelf convenience but remained vulnerable to thrombosis, infection, and neointimal hyperplasia resulting in stenosis [120].

### 6.2. Biologic Matrices

Biologic matrices aimed to go beyond simply structural support by also providing remodeling ability. Eggshell membrane (ESM) is a widely available and high biocompatible biomaterial with promising uses in medicine. Sun et al. [121] investigated its use in heparin-coated and non-coated vascular patches and discovered that ESM patches supported endothelialization while heparin reduced mural thrombus formation. This demonstrated that ESM could serve as a potential vascular patch material when surface chemistry is optimized. Similarly, Lu et al. [122] investigated the use of pulmonary visceral pleura (PVP) from porcine lungs in the development of a venous vascular patch. After glutaraldehyde cross-linking to reduce immunogenicity, PVP patches resulted in non-thrombogenic surfaces, neoendothelialization, and an actively organizing neomedia. However, the short duration of the study, lasting only 4 months, limits assessment of long-term outcomes. Together, ESM and PVP highlight some rather unconventional biologic tissues that demonstrate viable integration into vasculature patch design. Nevertheless, long-term durability remains a limitation along with processing consistency, opening the door for alternatives to emerge that combine the consistency of engineered scaffolds with biologics capable of remodeling.

### 6.3. Tissue-Engineered Patches

Engineered extracellular matrices and biopolymer composites improve reproducibility over natural biologic matrices while retaining remodeling potential [123]. One strategy has been through decellularization and seeding of human vascular tissue with hematopoietic stem cells (HSCs), which preserves the ECM, and HSCs infiltrate and repopulate the matrix [124]. In animal trials these constructs showed regeneration of both neoendothelialization and neomedia within 2 months of implant, producing tissue that closely resembled the native artery [124]. Similar ideologies have been used with porcine decellularized matrices and seeded with autologous cells. In porcine carotid arteries, these patches promoted neoendothelialization and limited neointimal hyperplasia [125]. More complex derivatives have been developed, such as drug delivery systems integrated with bilayer decellularized porcine pericardial patches [126]. The dual-layer matrix consisted of a lower decellularized ECM and an upper collagen layer with adipose stem cell exosomes. This bilayer provided structural rigidity due to the decellularized ECM and controlled delivery of adipose exosomes [126]. While this study focused on addressing diabetic mice, it exemplifies how scaffold architectures can be engineered to provide both structural vascular support and deliver biologically active cargo.

### 6.4. Biologically Active Patches

A lack of biologic interactions in early synthetic patches, such as ePTFE and Dacron^®^, left them susceptible to thrombosis, infection, and subpar endothelialization. Engineered biologically active patches expand upon the prior synthetics to retain durability and reproducibility, but are modified to improve hemocompatability, long-term integration with native vessels, and endothelialization. One example of this is coating ePTFE with polyurethane and polyurethane nanoparticles (PU/PU-NPs) [127]. This modification produced a textured patch lumen similar to native tissue topography, eliminated platelet adhesion, and supported endothelial progenitor cell adhesion and proliferation [127]. After implantation in mouse abdominal aortas, the PU/PU-NP patches remained patent and endothelialized at 30 days, whereas unmodified ePTFE stenosed within 48 h [127]. A complimentary approach used biodegradable polyhydroxybutyrate valerate/polycaprolactone (PHBV/PCL) scaffolds with covalently bound arginine-glycine-aspartate (RGD) peptides, a ligand for integrins, to promote bioactive molecule-induced repopulation by host cells [128]. By presenting bioactive ligands such as RGD, endothelial cell adhesion and proliferation is enhanced. Implanted in mouse abdominal aortas, these patches remained patent for up to 12 months and showed organized neotissue formation and resistance to calcification [128]. These studies demonstrate the impact of re-engineering and repurposing synthetic scaffold technology to incorporate bioactive properties, rather than simply inert fillers. Still, even with the successes of this generation of patches, they remain reactive rather than proactive. They integrate better with host tissues but cannot detect or report complications or signs of patch failure. Thus, future generations should continue expanding technology to address the need for a proactive vascular patch model.

### 6.5. Smart Patches

Smart patches represent the next generation by integrating functional bioelectronics (thin, flexible electronics) to provide proactive, real-time physiologic monitoring. One novel concept includes a piezoelectric patch based on aluminum nitride (AlN) that converts pulsatile wall motion into electrical signals, enabling continuous measurement of hemodynamic changes [129]. By using piezoelectric compounds that naturally generate electrical signals upon movement, the limitations of MEMS are bypassed. MEMS use a pressure based-sensor but require a voltage supply and electronic components that alter patch structure [129]. Tang et al. [130] further advanced piezoelectric vascular monitoring by engineering an implantable vascular electronic system consisting of a piezoelectric sensor, a tensile sheath to immobilize the piezoelectric sensor, and a wireless module for data transfer [130]. Importantly, the tensile sheath is elastic and can conform and grow with the corresponding vessel, allowing for pediatric or growing vessel applications [130]. In mouse and rabbit models, the system reported blood pressure, heart rate, and respiratory waveforms over a 10-week duration [130]. Collectively, smart patches mark the next generation of improvement and open the future to interactive, self-monitoring implants that continuously monitor hemodynamics and allow for proactive treatments. They offer the potential for early identification of complications such as thrombosis, neointimal hyperplasia, and stenosis before clinical failure.

**Table 5:** Representative patch technologies, key innovations, clinical limitations, and translational progress.

Device/Material	Innovations	Clinical Limitations	Preclinical/Clinical Trials
Structural Patches - Bovine pericardium - ePTFE - Dacron^®^	Provide mechanical reinforcement for repairs. ePTFE and Dacron^®^ offer off-the-shelf convenience.	Bovine pericardium can calcify and lacks ability to remodel or adapt to cardiovascular environment. ePTFE and Dacron^®^ are susceptible to thrombosis, infection, and neointimal hyperplasia resulting in stenosis.	[122]
Biologic Matrices - Egg Shell Membrane (ESM) - Pulmonary Visceral Pleura (PVP)	Ability to support endothelialization and resist thrombosis.	Durability and consistency are lacking.	[120,121,126]
Tissue-Engineered Patches	Combine the benefits of biologic remodeling with improved consistency by decellularizing human or porcine vascular tissue to be repopulated with stem cells. Some designs added additional controlled drug delivery.	Still experimental.	[124,125,131]
Biologically Active Patches - PU/PU-NP - PHBV/PCL	Modification of earlier ePTFE and Dacron^®^ scaffolds with polyurethane or RGD peptides to improve cell adhesion, prevent platelet adhesion, and resist calcification.	Inability to monitor the vascular environment.	[127,128]
Smart Patches - Piezoelectrics - Micro-electromechanic systems (MEMS)	Integrate modern biosensor technology into patches to provide continuous real-time physiological monitoring, providing a proactive approach to cardiovascular care.	Still in preclinical development and experimental stages.	[129,130]

## 7. Other Cardiovascular Implants

Beyond vascular and valvular devices, several other cardiac devices and cardiovascular implants play essential roles in rhythm management, circulatory support, and cardiac telemetry. The evolution of pacemakers, defibrillators, left atrial appendage occlusion (LAAO) devices, LVADs, and implantable cardiac telemetry systems demonstrates how materials innovation and convergence of nanotechnology and bioelectronics can be used to improve cardiovascular interventions.

### 7.1. Implantable Cardioverter-Defibrillators/Pacemakers

First developed for the management of bradyarrhythmias in 1958, early pacemakers provided right ventricular apical pacing via transvenous or epicardial leads connected to a subcutaneously implanted power source/generator [132,133]. However, the soon discovered downfall of early pacemakers was due to dyssynchrony between the right ventricle and left ventricle which caused increased risk of LV dysfunction, atrial fibrillation, and heart failure exacerbation [132]. Advancements included dual-chamber pacing which improved the synchronized actions between the RV and LV [134]. More recently, conduction system pacing via pacing of the His bundle and left bundle branch (LBB) has been found to preserve synchronous ventricular activation and reduce the risk of pacing-induced cardiomyopathy seen with asynchronous pacing methods [132]. Additionally, lead-related issues such as venous obstruction, infection, and tricuspid regurgitation led to the development of leadless pacemakers [134]. Altogether, advancements in symptomatic bradyarrhythmia treatment via pacemaker have demonstrated lowered mortality and morbidity in patients with heart failure, as shown through several clinical trials such as COMPANION, CARE-HF, MADIT-CRT, and the RAFT trial [134]. For patients with ischemic heart disease and non-ischemic cardiomyopathy, implantable cardioverter-defibrillators (ICDs) were developed in the 1970s as a method of treating survivors of sudden cardiac arrest [132]. Early ICDs used transvenous leads to monitor heart rhythm and deliver an electrical shock if arrhythmia was detected [132]. However, similar to early pacemakers, complications associated with transvenous leads prompted the development of subcutaneous ICDs, which reduced lead-related complications while maintaining comparable efficacy in the termination of arrhythmias, as evidenced by the PRAETORIAN and ATLAS clinical trials [134]. More recent advancements include the extravascular ICD, which used a substernal lead placed through a small subxiphoid incision to sit in front of the heart [132]. Advantages of extravascular ICDs include a lower shock energy needed due to proximity to the heart, no intravascular leads, and the ability to provide pacing for bradycardia that subcutaneous ICDs cannot, although clinical data supporting efficacy and long-term viability are still lacking [134].

### 7.2. Left Atrial Appendage Occlusion

Atrial fibrillation (AF) is the most common arrhythmia in the United States, affecting more than 6 million adults, and accounts for a large increase in stroke risk due to thrombus formation in the left atrium [135]. Standard AF therapy includes oral anticoagulation with warfarin or direct oral anticoagulants (DOACs), but for populations in which anticoagulation is contraindicated, such as the elderly, individuals with fall risk, or those with renal failure, alternatives such as LAAO devices are exceedingly useful [135]. The WATCHMAN^®^ by Boston Scientific is one of three options for LAAO, and the only FDA-approved LAAO for stroke prevention in non-valvular AF. It uses a self-expanding nitinol frame delivered via transseptal access [135,136]. By occluding the left atrial appendage, LAAOs prevent the development of thrombi within the appendage that typically occur due to hemostasis, thus reducing the risk of thrombus formation and stroke. Efficacy and safety in the PROTECT-AF and PREVAIL trials show that the WATCHMAN^®^ was non-inferior to warfarin at reducing stroke, systemic embolism, and cardiovascular death [135,136]. Other options, such as the Amplatzer Amulet^®^ and LARIAT^®^, do not have FDA approval for use in the U.S. but are used in other countries such as Europe [135]. The Amplatzer Amulet^®^ is also made of nitinol and data from usage outside the U.S demonstrate success rates exceeding 95% while complication rates fall below 2% [135]. The LARIAT^®^ uses a different method than WATCHMAN^®^ and Amplatzer Amulet^®^ by using a pretied suture to tie-off the LAA via combined transseptal and epicardial access; safety concerns arise due to a complication rate of roughly 10% [135,136]. Nevertheless, LAAOs provide a viable alternative treatment option to standard anticoagulation in AF patients, though anticoagulation remains first line for stroke prevention. The 2015 ACC/HRS/SCAI overview highlights the use of LAAOs in carefully selected patients where anticoagulation is contraindicated, but it stresses the need for specialized centers and training [136].

### 7.3. Left-Ventricular Assist Devices

Heart failure (HF) affects roughly 6.7 million people, with projections of 8.5 million by 2030 [137,138]. In advanced heart failure, which is characterized by severe and incapacitating symptoms at rest, LVADs provide the means of treatment by helping pump blood throughout the body [138]. LVADs serve a dual role based on the clinical needs of the patient and their specific disease course. For example, LVADs can be used for temporary ventricular assist in patients who do not have innate ventricular function while they wait for heart transplant [138]. Likewise, LVADs can also serve as permanent treatment options for those who are ineligible for heart transplantation [138]. Like most other cardiac devices, LVADs underwent generational innovations that improved clinical outcomes and patient quality of life, whether that be in transition to heart transplant or permanent therapy. The first internal LVAD, HeartMate^®^ XVE, mimics native heart function using a pulsatile electric pump with both systolic and diastolic phases [138,139,140]. In the Randomized Evaluation of Mechanical Assist Treatment for Congestive Heart Failure (REMATCH) trial, the HeartMate^®^ XVE was compared against the non-transplant standard therapy, which was medication only and consisted of Beta-blockers, spironolactone, and ACE inhibitors [138,140]. The results from this trial showed a significantly increased 2-year survival in the LVAD cohort, which was 23% compared to the 8% seen in standard therapy [138]. The success was short-lived, though, as the HeartMate^®^ XVE lacked durability due to the complexity of its internal components, with near-universal failure of devices between 18 and 24 months [138]. The durability concerns of the HeartMate^®^ XVE pushed researchers toward the development of the HeartMate^®^ II, which was a continuous pump [138,140]. From this point on, all subsequent innovations and LVAD devices have been continuous pumps because of the simplicity of the internal components that make for the excellent durability and lifespan of the device. Driven by an internal rotor, the HeartMate^®^ II and HM3 LVAD mark the latest advancements in LVAD technology. The key difference is that the HeartMate^®^ II uses mechanical bearings to support the rotor whereas the HM3 LVAD uses magnetically levitated rotor [138,140]. The MOMENTUM 3 trial compared the HeartMate^®^ II and HM3, demonstrating superiority in the HM3 and is thus the only LVAD used in the United States today [138,140].

### 7.4. Smart Cardiac Telemetry Implants

The foundation of future advancements of cardiac telemetry lies in the advancement of bioelectronics designs specifically for cardiac monitoring. Several implantable devices have been developed for cardiac telemetry, including the Medtronic Chronical, St. Jude Medical HeartPOD, Vectorious Medical Technologies left atrial pressure sensor (LAP), St. Jude Medical CardioMEMS, and the Endotronix Cordella [141]. Common functionalities of these sensors include their ability to remotely transmit cardiac data such as LAP, heart rate, systolic/diastolic ventricular pressures, temperature, intracardiac electrograms, and pulmonary artery pressure [141]. Despite their success in cardiac monitoring, concerns regarding the rigidity of these devices and inability to move with the surrounding epicardium remain, which can result in patient discomfort as well as tissue damage due to fibrosis or scarring [141]. To overcome this biomechanical mismatch, the innovation of soft and stretchable substrates that conform to cardiac motion enable integration without inducing inflammatory responses is necessary to improve clinical outcomes. Thus, the future of cardiac telemetry should focus on furthering innovation in both hemodynamic monitoring and integration of the system with the fluidity of the human body.

## 8. Clinical Translation Challenges of Smart Cardiovascular Implants

### 8.1. Long-Term Reliability

Long-term reliability remains one of the most significant barriers limiting clinical translation of smart cardiovascular implants [142]. In addition to structural integrity, smart cardiovascular implants must also demonstrate longevity in sensor accuracy, signal stability, and data security [143,144]. Unlike conventional passive devices, smart implants rely on integrated electronic components that are continuously exposed to cyclic mechanical loading, pulsatile blood flow, inflammatory processes, and tissue remodeling. These factors may contribute to sensor drift, conductor fatigue, biofouling, fibrotic encapsulation, or degradation of the device/tissue interface. Failure of sensing systems may lead to inaccurate physiologic measurements, delayed detection of complications, or inappropriate clinical decision making. Consequently, future development efforts must focus on improving sensor robustness, minimizing biological fouling, and establishing long-term in vivo validation protocols capable of demonstrating sustained functionality under clinically relevant conditions [143,144].

### 8.2. Patient Safety and Biocompatibility

The integration of flexible electronics, wireless telemetry systems, and embedded sensors introduces additional patient safety considerations beyond those encountered with traditional cardiovascular implants [145]. Smart implants must also demonstrate that embedded electronic components do not adversely affect surrounding tissues through heat generation, electromagnetic interference, material degradation, or unintended electrical stimulation. Furthermore, device failure modes must be carefully evaluated to ensure that loss of sensing capability or wireless communication does not compromise the primary therapeutic function of the implant [146]. For example, a sensor-integrated stent must continue to maintain vessel patency even if electronic systems fail. As a result, long-term biocompatibility testing and chronic implantation studies will be essential components of future smart implant development and regulatory evaluation [143].

### 8.3. Regulatory Approval Considerations and Cybersecurity

Regulatory approval represents one of the more complex barriers facing the clinical translation of cardiovascular implants and is even more complex for smart implants [21]. Unlike conventional devices, smart implants combine implantable biomaterials with sensors, microelectronics, and wireless communication systems [147]. As a result, these technologies must satisfy regulatory requirements applicable to both implantable medical devices and digital health systems. The U.S. Food and Drug Administration (FDA) employs a risk-based regulatory framework in which implantable cardiovascular devices are generally subject to the most rigorous levels of evaluation due to their life-sustaining nature and potential for significant patient risk [143]. For smart cardiovascular implants, regulatory assessment extends beyond traditional measures of mechanical safety and biocompatibility to include software validation, cybersecurity protection, wireless communication reliability, data integrity, and long-term system performance. Consequently, developers must generate extensive preclinical evidence demonstrating hemocompatibility, cytotoxicity, immunogenicity, chronic biocompatibility, fatigue resistance, electrical safety, and electromagnetic compatibility before entering clinical studies [143]. In addition, cybersecurity has emerged as a particularly important regulatory consideration for smart cardiovascular devices. Das et al. emphasized that implantable electronic devices increasingly rely on wireless telemetry, cloud-based data storage, and remote monitoring infrastructures, which create potential vulnerabilities that could affect both patient privacy and device functionality [144]. Several FDA safety communications involving commercially available cardiac implantable electronic devices have demonstrated that cybersecurity concerns are no longer theoretical but represent an active component of regulatory oversight [144]. As such, future smart cardiovascular implants will likely require robust encryption, secure authentication protocols, software verification, and continuous post-market security monitoring in addition to long-term device performance, safety, and durability regulations [143,144]. Additionally, regular software updates during the developmental stages is believed to improve device ecosystem security and updates should extend into the post-implantation lifetime of the device—necessitating communication with the patient regarding the need for such device updates [144].

## 9. Conclusions and Future Perspectives

Although cardiovascular implants encompass diverse device categories and clinical applications, their evolution has been driven by a common set of biological, engineering, and translational challenges. Across stents, heart valves, vascular grafts, cardiac patches, and implantable bioelectronic systems, the field has progressively transitioned from passive structural replacements toward biologically interactive, regenerative, and increasingly intelligent therapeutic platforms. This evolution reflects a growing recognition that long-term clinical success depends not only on restoring anatomy or hemodynamic function, but also on achieving durable integration with the dynamic cardiovascular microenvironment.

Several recurring challenges have consistently shaped the development of successive generations of cardiovascular implants. Thrombosis, chronic inflammation, inadequate endothelialization, compliance mismatch, structural degeneration, and insufficient tissue integration remain major barriers to long-term device performance. Despite substantial technological advances, a persistent trade-off continues to exist between durability and biological functionality. Synthetic and mechanically robust implants often provide superior structural longevity but may suffer from limited biological integration, whereas biologic and tissue-engineered constructs offer improved biocompatibility yet remain constrained by structural instability, manufacturing complexity, and inconsistent long-term outcomes. Resolving these competing requirements remains one of the central challenges in cardiovascular implant engineering.

Importantly, the trajectory of cardiovascular implant development increasingly reflects a paradigm shift from structural replacement toward functional restoration, tissue regeneration, and dynamic host/device interaction. Advances in biomaterials, tissue engineering, pharmacologic modulation, regenerative medicine, and implantable bioelectronics have expanded the capabilities of cardiovascular implants far beyond mechanical support alone. Emerging technologies such as smart biomaterials, soft bioelectronics, wireless biosensors, nanotechnology, and artificial intelligence are enabling real-time physiological monitoring, adaptive therapeutic responses, and personalized treatment strategies.

Looking forward, the future of cardiovascular implants will likely be defined by the convergence of regenerative biology, advanced materials science, bioelectronics, and data-driven medicine. Next-generation implants are expected to become increasingly patient-specific, biologically integrated, and capable of continuously sensing, adapting to, and interacting with their surrounding environment. Continued progress will require not only technological innovation but also solutions to critical translational challenges involving long-term reliability, scalability, manufacturability, regulatory approval, and clinical implementation. Ultimately, the successful integration of these disciplines holds the potential to transform cardiovascular implants from passive replacement devices into intelligent therapeutic systems capable of restoring and maintaining cardiovascular function throughout the patient’s lifetime.

## Figures and Tables

**Table 1:** Pathophysiological challenges, clinical demands, and representative cardiovascular implant technologies across major cardiovascular diseases.

Disease Category	Pathophysiological Challenge	Clinical Demand	Representative Implant Technologies
Ischemic Heart Disease	Coronary artery occlusion and ischemia	Restore perfusion, prevent restenosis, maintain vessel patency	Bare-metal stents, drug-eluting stents, bioresorbable scaffolds, smart stents [5]
Valvular Heart Disease	Stenosis and regurgitation	Restore unidirectional blood flow, durability, hemocompatibility	Mechanical valves, bioprosthetic valves, polymeric valves, TAVR, TEHVs [7]
Cardiac Arrythmias	Abnormal electrical conduction	Rhythm monitoring and correction	Pacemakers, implantable cardioverter-defibrillators, electrophysiologic mapping systems [8]
Myocardial Injury	Loss of viable myocardium and impaired contractility	Tissue repair, regeneration, functional support	Cardiac patches, tissue-engineered constructs, bioelectronic patches [9]
Vascular Disease/Aneurysms	Vessel occlusion, degeneration, or structural failure	Restore blood flow, mechanical support, compliance matching	Synthetic grafts, autologous grafts, tissue-engineered vascular grafts, smart vascular grafts [10]

## Data Availability

No new data were created or analyzed in this study. Data sharing is not applicable to this article.
